# A multi‐dimensional assessment of financial hardship of cancer patients using existing health system data

**DOI:** 10.1002/cam4.6731

**Published:** 2023-11-21

**Authors:** Wen You, Asal Pilehvari, Ruoding Shi, Wendy Cohn, Christina Sheffield, Philip I‐Fon Chow, Becca Anne Krukowski, Roger Anderson

**Affiliations:** ^1^ University of Virginia Comprehensive Cancer Center Charlottesville Virginia USA

## Abstract

**Background:**

Existing financial hardship screening does not capture the multifaceted and dynamic nature of the problem. The use of existing health system data is a promising way to enable scalable and sustainable financial hardship screening.

**Methods:**

We used existing data from 303 adult patients with cancer at the University of Virginia Comprehensive Cancer Center (2016–2018). All received distress screening and had a valid financial assistance screening based solely on household size‐adjusted income. We constructed a composite index that integrates multiple existing health system data (Epic, distress screening, and cancer registry) to assess comprehensive financial hardship (e.g., material conditions, psychological responses, and coping behaviors). We examined differences of at‐risk patients identified by our composite index and by existing single‐dimension criterion. Dynamics of financial hardship over time, by age, and cancer type, were examined by fractional probit models.

**Results:**

At‐risk patients identified by the composite index were generally younger, better educated, and had a higher annual household income, though they had lower health insurance coverage. Identified periods to intervene for most patients are before formal diagnosis, 2 years, and 6 years after diagnosis. Within 2 years of diagnosis and more than 4 years after diagnosis appear critical for subgroups of patients who may suffer from financial hardship disparities.

**Conclusion:**

Existing health system data provides opportunities to systematically measure and track financial hardship in a systematic, scalable and sustainable way. We find that the dimensions of financial hardship can exhibit different patterns over time and across patient subgroups, which can guide targeted interventions. The scalability of the algorithm is limited by existing data availability.

## 1 INTRODUCTION

Cancer treatment costs have increased despite more effective treatments, driving many cancer patients and their families into financial hardship.^1^, ^2^, ^3^ Previous research has mostly focused on objective metrics (i.e., out‐of‐pocket expenses (OOP) and income). However, current conceptualizations of financial hardship have acknowledged a need for more comprehensive assessment of objective and subjective distress related to cancer treatments: material conditions, psychological responses, and coping behaviors.^4^

Material conditions refer to patient's direct (e.g., OOP)^5^ and indirect costs (e.g., productivity loss, employment interruption, and related bankruptcies).^7^, ^8^, ^9^ Psychological responses refers to patient's treatment‐related mental distresses (e.g., fear of financial burden, worry about side effects, fear of cancer recurrence).^10^ Coping behaviors are actions taken by patients to ease their financial burden (e.g., skipping/delaying treatments, forgoing activities in life that result in negative treatment outcomes and reduction in quality of life).^5^, ^6^, ^7^ Given the multifaceted nature of financial hardship and its negative outcomes, it is important to devise a more comprehensive measurement that captures those separate yet related dimensions.

Currently available multidimensional measures of financial hardship in cancer patients^8^, ^9^, ^10^ have limitations that hinder their scalability, sustainability, and effectiveness. First, these measures require data beyond the information regularly housed in the health system, which poses a significant implementation barrier due to increased patient response burden and could disrupt clinic workflow.^7^, ^11^ Second, they mostly assess financial hardship cross‐sectionally, even though financial hardship levels may fluctuate over time and across different dimensions.^12^ Effective financial hardship interventions should be delivered at the right time, ideally before a patient's financial hardship worsens.^13^, ^14^, ^15^, ^16^

An ideal way to proactively assess and monitor financial hardship levels is to leverage existing data sources present at most health systems.^17^ Specifically, clinic records contain information that can be used to measure material condition hardship over time (i.e., employment, occupation, income, insurance, payment histories). Furthermore, all accredited U.S. cancer centers are required to screen for psychological distress over time,^18^ which can be used for measuring psychological responses. Finally, cancer centers commonly maintain a registry that contains information on treatment‐related coping behaviors (e.g., diagnosis, prognosis, progression, treatment plan, treatments completed).

The purpose of this study is to develop a composite measure of financial hardship (overall and by dimension) using these existing health system data sources to understand how financial hardship may change over time. The ultimate goal of this study is to improve financial hardship screening by developing a low‐cost, scalable, and sustainable method of assessment that can advance our understanding of when, and how often, to screen for financial hardship in cancer patients.

## 2 METHODS

### 2.1 Data and study sample

We used three sources of data from the University of Virginia Comprehensive Cancer Center health system from 2016 to 2018: (1). Material condition: electronic health records, maintained in Epic, of patients' healthcare utilization, payment history, insurance status, and financial assistance eligibility (assessed solely by household size adjusted income); (2) Psychological responses: distress screening data, collected during patients' oncology visits; and (3) Coping behaviors: cancer registry data, which contains cancer patients' clinical characteristics (e.g., cancer diagnosis/stage, treatment adherence indicators, and prognosis). The study was approved by the University of Virginia Institutional Review Board for Health Sciences Research (IRB‐HSR#22686).

**FIGURE 1:**
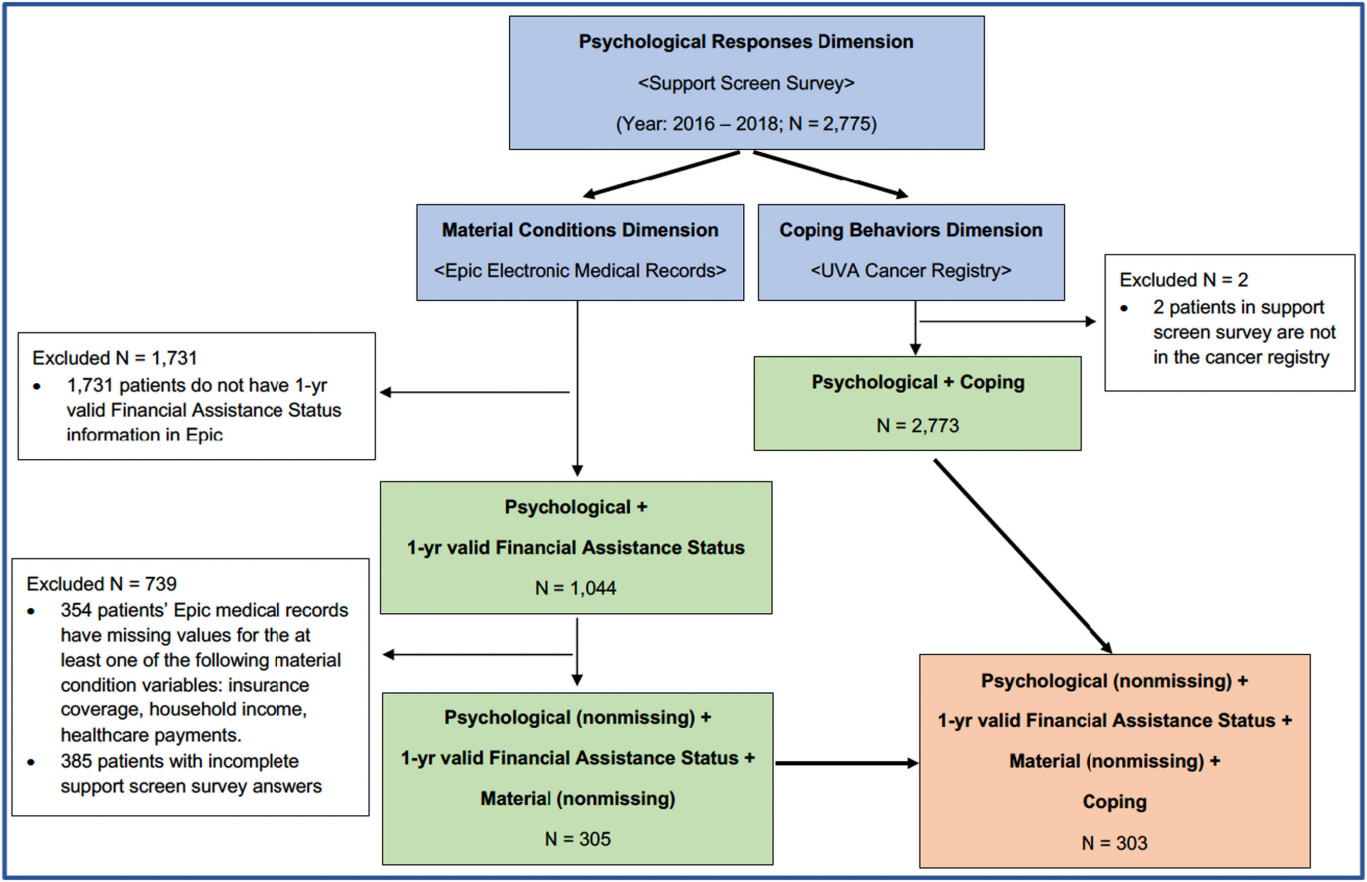
Data Sources and Sample Selection.

We only included patients who had data pertaining to each financial hardship dimension (Figure 1). Among the three dimensions, psychological response was most limited because it was only widely implemented starting in 2016 (*N* = 2,775 adult cancer patients). Furthermore, patients in our sample also needed to have financial assistance status informed by the current single‐dimension screening practice. To ensure different data sources form a meaningful assessment, we required assessments at least once per year, excluding 1,731 patients.* Among the remaining 1,044 patients, 741 had missing data. The final analytical sample consists of 303 eligible patients with complete data.

### 2.2 Measures

#### 2.2.1 Material condition dimension

Material condition variables were defined as follows: (1) *Income*: annual household income before taxes. (2) *OOP/Income*: percentage of household annual income spent in medical care that is not covered by insurance. (3) Health insurance status: percentage of medical care spending that is covered by insurance (*Coverage*), indicator for uninsured (*Uninsured*), and indicator for Medicaid recipient (*Medicaid*). (4) Financial assistance (FA) eligibility and levels: eligibility (*FA status*) and specific levels of financial assistance qualified (*FA levels*). The UVA Health System FA screening is based solely on the comparison of a patient's household income (adjusted by number of dependents) and the 400% federal poverty level (FPL). If adjusted income is below 400% FPL, the patient is eligible for FA. The specific amount of FA depends on the severity of poverty.†

#### 2.2.2 Psychological responses dimension

**FIGURE B1:**
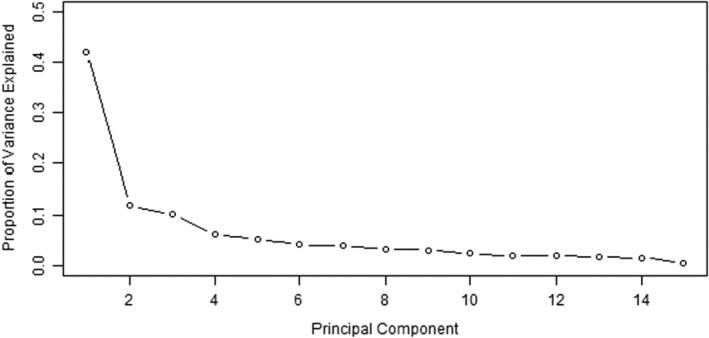
Screen plot of cumulative variance explained by principal components.

Psychological responses were measured through a  15‐item distress screener (5‐point Likert scale ranging from “not a problem” to “very severe problem”). To reduce the dimensionality of the dataset and to minimize redundancy,^19^ we conducted a principal component analysis (PCA) and identified four components that explain >70% of the total variance of the distress screening data (Figure B1). Those principal components (i.e., affect, finances, energy, and treatment) served as the four psychological response indicators to be used to measure the psychological responses dimension (Appendix 2; Table B1).

#### 2.2.3 Coping behaviors dimension

We extracted patients' treatment noncompliance information associated with immunotherapy, radiation, chemotherapy, and hormone therapy from the cancer registry. If any recommended cancer treatment was documented as refused and was not due to other medical complications, the associated treatment noncompliance indicator is set to 1. Additionally, using cancer diagnosis dates and disease‐specific treatment expectations from Rapid Quality Report System,‡ we constructed a binary variable identifying cases in which recommended treatments were not delivered within the expected timeline.

### 2.3 Constructing a composite index of financial hardship

**FIGURE 2:**
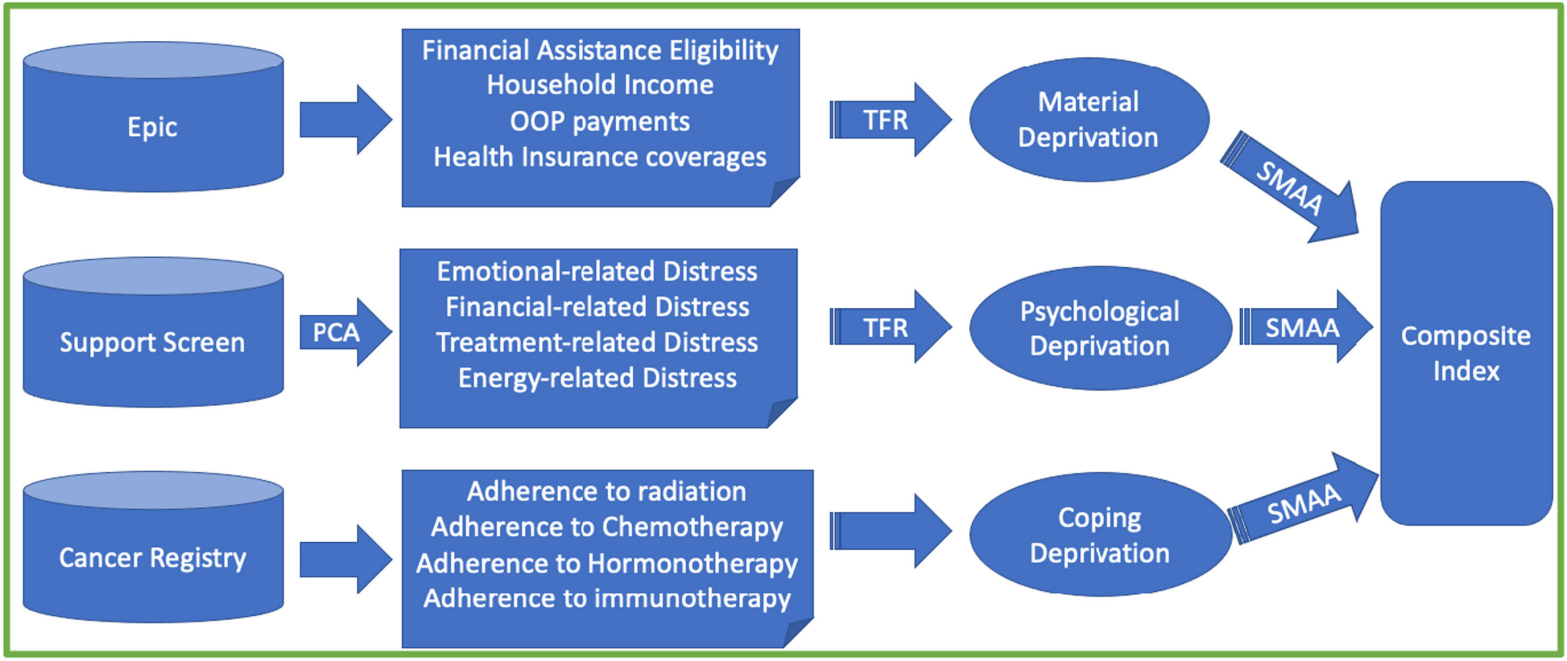
Composite Index Algorithm Illustration. PCA, principal component analysis; SMAA, stochastic multi‐criteria acceptability analysis; TFR, totally fuzzy and relative approach.

Figure 2 displays how we constructed the index despite three challenges: scale differences (e.g., dollars and percentages), aggregating variables within a dimension, and calculating the dimension‐specific weights.

#### 2.3.1 Scale differences

We used the totally fuzzy and relative approach (TFR)^20^ to convert variables to a uniform scale between 0 and 1, where 0 indicates no hardship in that indicator or domain and 1 indicates the highest degree of hardship (see Appendix 1).

#### 2.3.2 Variables aggregation within dimension

To generate a dimension‐specific index, we used deprivation‐weighted average as the aggregating method,^21^ which assigns larger weights to those experiencing hardship using the log of the sample mean inverse.

#### 2.3.3 Dimension‐specific weight calculation

To create a composite index from the three dimensions, we applied stochastic multi‐criteria acceptability analysis (SMAA) to have robust weight estimates and address the uncertainty surrounding the weight choices.^22^, ^23^ During each simulation, SMAA draws weight vectors from a uniform distribution within feasible weights and calculate a composite index that is a weighted average across all three dimension‐specific scores.§

## 3 STATISTICAL METHODS

We conducted Kruskal–Wallis rank sum tests to examine financial hardship differences by age and time since initial diagnosis. We used a two‐sample *t*‐test to compare patient characteristics eligible for financial assistance under the current standard with those of high‐risk patients identified by the composite score. Consistent with the current financial hardship screening literature,^8^, ^26^ we used the sample median to identify patients at risk of financial hardship. Within high‐risk patients identified by the composite index, we also tested the difference between FA‐eligible and FA‐ineligible subgroups of patients based on age and time since diagnosis.

Lastly, we built a predictive model to analyze the financial hardship dynamic patterns over cancer survivorship. Since the financial hardship scores are bounded between 0 and 1, we estimated fractional probit models by regressing the dimensional scores and overall composite index score on patient demographics (e.g., age, gender, race, ethnicity, household income, and education) and clinical characteristics (i.e., cancer types, cancer stage, time after initial cancer diagnosis, and interaction between types of cancer and cancer stage). Statistical analyses were conducted using R^27^ and Stata 16.^28^

## 4 RESULTS

### 4.1 Summary statistics

The sample was primarily Caucasian, female, non‐Hispanic, married, and had at least some college education (Table 1), similar to a general population of U.S. cancer survivors. Slightly over half were 65+ years old and had early to mid‐stage cancers. Breast cancer was the most common cancer diagnosis followed by lung and other (e.g., rectal/skin/endometrial cancer).

**TABLE 1:** Summary statistics of demographics and cancer‐related characteristics of the study sample.

Variables	Description	Overlapping sample^a^ (*n* = 303)
Highest education level	Less than high school	24 (8.3)
	High school degree	69 (23.8)
	Some college or higher	197 (67.9)
Stages of cancer	Early to mid‐stage (stage 0 to 2)	158 (54.5)
	Late stage (stage 3 and 4)	132 (45.5)
Marital status	Not married (single/divorced/widowed)	95 (31.4)
	Married	208 (68.6)
Ethnic group	Hispanic	1 (0.3)
	Not‐hispanic	302 (99.7)
Gender	Female	224 (73.9)
	Male	79 (26.1)
Race	African American	10 (3.3)
	Other (e.g., Asian, Native Hawaiian)	7 (2.3)
	White or Caucasian	285 (94.4)
Type of cancer	Breast	104 (34.3)
	Colon	27 (8.9)
	Lung	86 (28.4)
	Other (e.g., rectal/skin/endometrial cancer)	86 (28.4)
Age group	18–44	20 (6.6)
	45–64	125 (41.3)
	65+	158 (52.1)

^a^ The overlapping sample included 303 patients with complete information from dimensions of material condition, psychological responses, and coping behaviors.

As shown in Table 2, on average, annual income was $69,513.30 with 1% out‐of‐pocket payments over income. All patients had health insurance, and, on average, 95% of payments were covered by health insurance. Only 10% had Medicaid coverage.

**TABLE 2:** Summary statistics of financial hardship variables and calibration methods used by dimensions.

Dimension	Variable	Description	Overlapping sample^a^ (*n* = 303)	Calibration methods
Material conditions	Income	Annual household income ($)	69,513.3 +/− 54,168.2	TFR for those with income ≤400% FPL
	OOP/Income	The ratio of OOP payment over household income (%)	1 +/− 3.5	TFR
	Coverage	% of payments covered by insurance	95 +/− 16.3	TFR
	Uninsured	0/1 indicator: if the person is uninsured *n* (%)	0 (0)^b^	Unchanged
	Medicaid	0/1 indicator: if the person receives Medicaid *n* (%)	30 (10)	Unchanged
	FA status	Eligible for FA but not received (*n* (%))	89 (29.4)	Unchanged
		Received FA (*n* (%))	15 (4.95)	Unchanged
		Not eligible for FA (*n* (%))	199 (65.67)	Unchanged
	FA levels	over 400% FPL	202 (66.7)	TFR (linear assignment)
		Charity	1 (0.3)	
		30%–55% FA	48 (15.8)	
		80%–95% FA	34 (11.2)	
		100% FA	18 (5.9)	
Psychological Responses	Emotion	0–1 scaled principal component 1: emotional distress	0.4 +/− 0.3	TFR
	Finances	0–1 scaled principal component 2: financial‐related distress	0.4 +/− 0.3	TFR
	Energy	0–1 scaled principal component 3: energy‐deprivation	0.5 +/− 0.3	TFR
	Treatment	0–1 scaled principal component 4: treatment‐related worry	0.5 +/− 0.3	TFR
Coping Behaviors	Immune	0/1 indicator: if the patient failed to adhere to immunotherapy	1 (0.3%)	Unchanged
	Radiation	0/1 indicator: if the patient failed to adhere to radiation	4 (1.32%)	Unchanged
	Chemo	0/1 indicator: if the patient failed to adhere to chemotherapy	10 (3.3%)	Unchanged
	Hormone	0/1 indicator: if the patient failed to adhere to hormone therapy	4 (1.32%)	Unchanged
	Timeline	0/1 indicator: if the patient failed to adhere to expected treatment timeframe	1 (0.3%)	Unchanged

Abbreviations: FA, financial assistance; FPL, federal poverty line; OOP, out‐of‐pocket; TFR: totally fuzzy and relative approach

^a^ The overlapping sample included 303 patients with complete information from dimensions of material condition, psychological responses and coping behaviors.

^b^ The patients in the overlapping sample all have some sort of health insurance.

Almost one‐third was eligible for FA but did not receive it (29.4%). Roughly two‐thirds (65.67%) were not eligible for FA. Only 4.95% were eligible for FA and actually received it. In the psychological responses dimension, energy deprivation and treatment‐related worries have slightly higher hardship levels as compared to emotional distress or direct financial worries (Table 2).¶ In the coping behaviors dimension, our data showed a high degree of treatment adherence. The largest nonadherence was chemotherapy treatment to which about 3.3% of the patients did not adhere.

### 4.2 Composite index and dimensional scores

**FIGURE 3:**
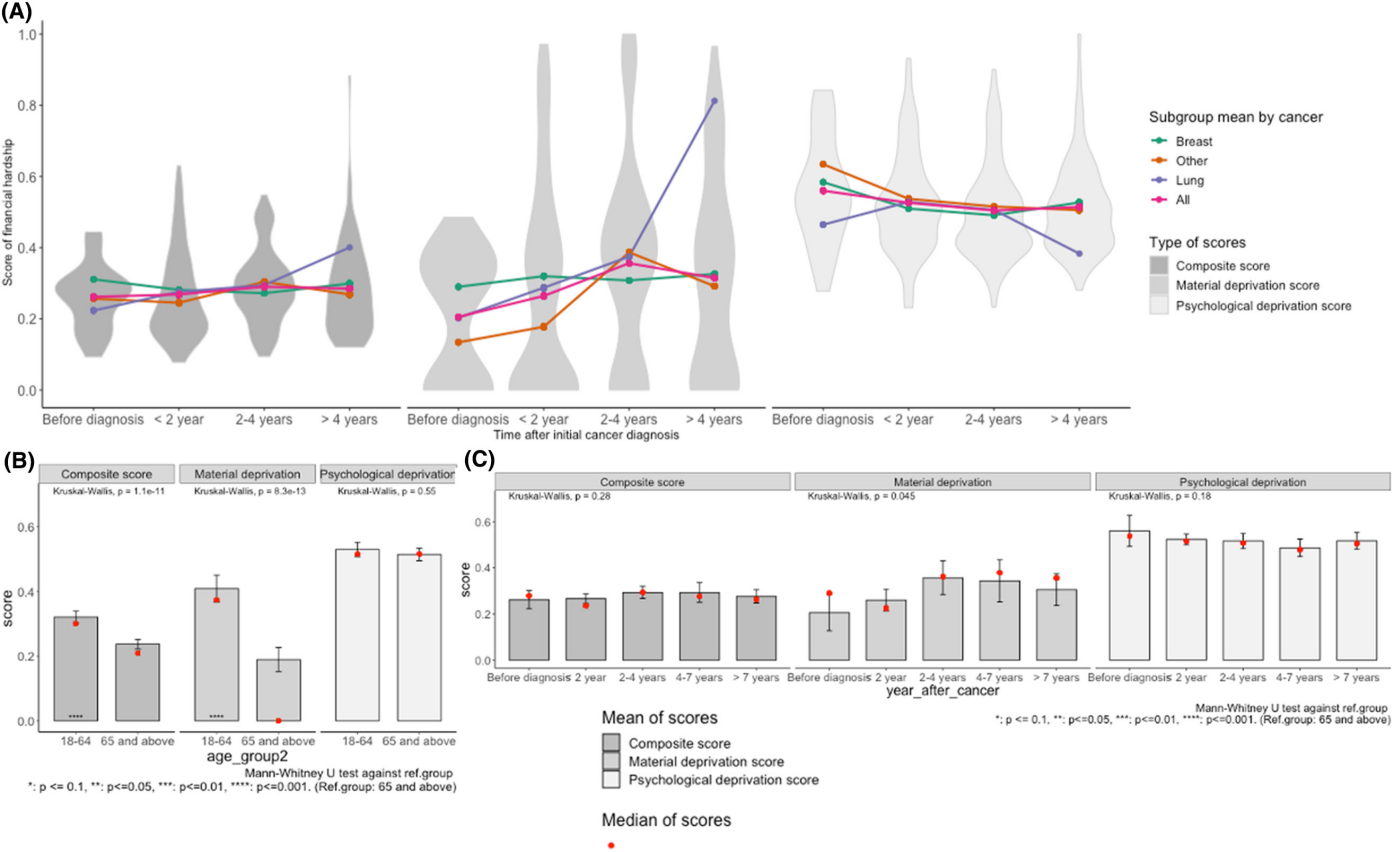
Multi‐dimensional financial hardship associated with cancer by cancer sites, stages, time after diagnosis and age group (unadjusted, overlapping sample, *N* = 303). The figure shows subgroup mean, median, and 95% confidence intervals of adjusted group means.

Figure 3A summarizes unadjusted financial hardship scores (i.e., composite scores, material condition and psychological responses dimension scores) for overall sample and by cancer type. The coping behaviors dimension is not presented due to low treatment noncompliance frequency in the data. Material hardship increased sharply before initial cancer diagnosis, peaked 4 years later, and then decreased across cancer types, except for lung cancer patients for whom the material hardship soared 4 years after diagnosis, consistent with high costs of advanced lung cancer treatments.^30^, ^31^ In contrast, psychological hardship peaked before diagnosis and stayed relatively consistent over time for the overall sample, but for lung cancer patients, it peaked around 2 years after diagnosis and decreased after 4 years.

The composite scores captured time‐trend differences in financial hardship domains, with slower increases of hardship levels until 4 years after diagnosis and slight decreases thereafter. In contrast, lung cancer patient composite hardship levels continued rising but at a slower rate. The composite score distributions also revealed severe financial hardship disparities at two time periods (i.e., more positive‐skewed): less than 2 years after the formal diagnosis and more than 4 years after the diagnosis. At each time point, a subset of patients experienced disproportionally high levels of overall financial hardship compared to the majority. Importantly, this is overlooked when only examining the single‐dimension scores. Material dimension scores showed a sizable portion of patients were not deprived at all (i.e., a high proportion of patients have material hardship score of zeros), while psychological dimension scores indicated all patients suffered distress to some degree. The composite score, by contrast, indicated most of the sample had minor to mild financial hardship, with a small portion experiencing extreme hardship.

Adults 18–64 years old had higher median hardship compared to those 65 or older (median score = 0.30 vs. 0.21, *p* < 0.001; Figure 3B), mainly due to material dimension differences. Median material hardship levels were significantly higher for patients 2+ years after formal diagnosis than before diagnosis (Figure 3C). However, psychological dimension median scores differed only between patients 4–7 years after diagnosis and before. Therefore, the overall composite scores did not show statistically significant differences across time since diagnosis.

### 4.3 Dynamic patterns of financial hardship over the course of cancer survivorship

**FIGURE 4:**
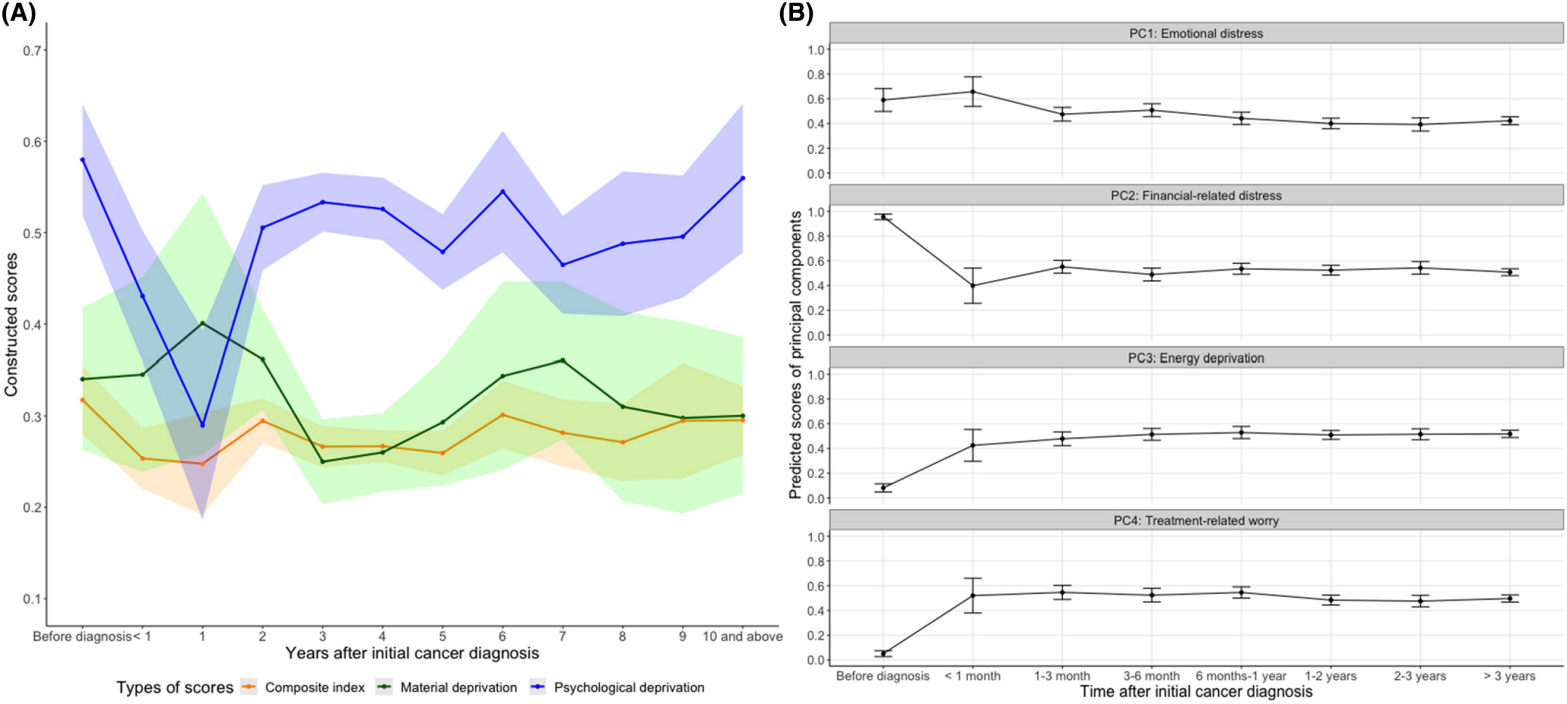
Dynamic patterns of financial hardship levels over the course of cancer survivorship. The figure shows point estimate and 95% confidence intervals in error bars or bands for adjusted group means.

The composite scores (in red) showed a relatively stable time trend, while dimensional scores (material condition score in green and psychological responses score in purple) fluctuated greatly (Figure 4A). The psychological responses dimension showed a high distress before cancer diagnosis, declining to half the level about a year after diagnosis, consistent with existing studies,^32^, ^33^, ^34^ while the material condition dimension slowly increased, peaking about a year after diagnosis. Between 1 and 5 years since diagnosis, psychological hardship increased sharply and plateaued slightly lower than before diagnosis, while material hardship decreased and reached its lowest level at around 3 years since diagnosis. Long‐term (10 years and above) psychological hardship levels are still predicted to remain high while the material hardship remains moderate.

Figure 4B highlights changes over time in each principal component of the psychological dimension. Emotional distress was particularly high before and within 1 month after diagnosis, then decreased. Financial‐related distress peaked before cancer diagnosis and decreased about 1 month after diagnosis. As expected, distress related to energy deprivation and treatment was close to zero before diagnosis, then increased rapidly in the first 6 months, and did not decline even among patients surviving more than 3 years.

### 4.4 Benefits of using the Composite Index Over the Current Screening Standard

We identified 151 patients at risk of financial hardship with composite index scores over the sample median (*At‐Risk*), while 101 patients were eligible for FA under the current financial screening standard (*FA‐‐Eligible*) (Table 3). Compared with the *FA‐Eligible* group, patients in the *At‐Risk* group tended to be younger, more likely to have a college education, have a higher household income, and have lower insurance coverage. We found no group differences statistically by cancer type.

**TABLE 3:** Characteristics of patients eligible for financial assistance and comparison groups identified by composite index.

Characteristics	Description	FA‐Eligible	At‐Risk	p‐Value^a^	At‐Risk & FA‐eligible	At‐Risk & FA‐ineligible	p‐Value^b^
Number of patients		101	151	–	90	61	–
Age	Age in years	63.71	60.58*	0.078	63.5	56.26***	0.001
OOP/income	The ratio of OOP payment divided by FPL income (%)	1.25	1.81	0.335	1.41	2.4	0.196
HH income	Household annual income	23,469.86	49,567.7***	<0.001	22,696.53	89,213.67***	<0.001
Insurance coverage	% of payments covered by insurance	97.78	91.98**	0.001	97.51	83.82***	<0.001
Cancer time	Days after the initial cancer diagnosis	1661.99	1499.08	0.526	1731.62	1150.26*	0.05
Medicaid (%)	0/1 indicator: if receives Medicaid	20	18	0.705	22	11*	0.077
Received FA (%)	0/1 indicator: if receives financial assistance	16	10	0.18	17	0***	<0.001
Late‐stage cancer (%)	0/1 indicator: if has late‐stage cancer	52	49	0.654	54	41	0.114
College (%)	0/1 indicator: if obtain some college education or higher	48	60*	0.088	48	76***	<0.001
Married (%)	0/1 indicator: if is married	57	64	0.331	57	74*	0.029
White (%)	0/1 indicator: if is white	94	91	0.324	93	87	0.215
Female (%)	0/1 indicator: if is female	71	77	0.332	73	82	0.208
Types of cancer							
Breast (%)	0/1 indicator: if has breast cancer	28	37	0.118	30	48*	0.032
Colon (%)	0/1 indicator: if has colon cancer	6	9	0.32	7	13	0.209
Lung (%)	0/1 indicator: if has lung cancer	35	28	0.256	33	20	0.059
Other (%)	0/1 indicator: if has other cancer	32	26	0.319	30	20	0.146

*Note*: The overall sample for this Table is 252 since there are 51 patients in the total 303 patient sample do not have relevant demographic and/or cancer clinical information recorded. To compare the characteristics of patients eligible for financial assistance under current mechanism with high‐risk patients identified by the composite score, this table reports means with asterisks (*, **, ***) indicate statistically significant differences in sample mean at the 5%, 1%, and 0.1% level based on two sample *t*‐test.

^a^ Patients in At‐Risk group have composite index score larger than sample median composite index score. The *p*‐values come from two sample *t*‐test between FA‐eligible group with At‐Risk group.

^b^ The *p*‐values come from *t*‐test between At‐Risk & FA‐eligible group and At‐Risk & FA‐ineligible group.

Similarly, among the 151 patients in the *At‐Risk* group, 90 patients were eligible for FA by the current standard (*At‐Risk & FA‐eligible*). We compared the remaining 61 patients in the At‐Risk group who were not eligible for FA (*at‐Risk & FA‐ineligible*) with those 90 patients who were eligible (Table 3). Those patients who were at‐risk but not eligible for FA (*At‐Risk & FA‐ineligible*) were younger, more likely to have a college education, had a higher household income, had a lower percentage of Medicaid insurance coverage, and had lower overall insurance coverage. Moreover, the *At‐Risk & FA‐ineligible* group tended to be in the early phases of their cancer survivorship, were more likely to be married, were more likely to have breast cancer, and were less likely to have lung cancer. These findings point to the vulnerable patient population that is overlooked if the financial hardship screening uses only material condition indicators.

## 5 DISCUSSION

The purpose of this study is to demonstrate a way to resourcefully utilize existing health system data to improve multi‐dimensional financial hardship screening that can advance our understanding of when, and how often to screen cancer patients. We found that financial hardship levels can exhibit different patterns across time and different dimensions.^35^ Based on our results, there may be three opportune periods to intervene when most cancer patients suffer from both material condition and psychological responses hardship: before diagnosis, 2 years after diagnosis, and 6 years after diagnosis. Furthermore, our findings indicate a small subset of patients disproportionally suffer financial hardship at two time periods: less than 2 years after diagnosis and more than 4 years after diagnosis. Targeted screening during those periods may be particularly important for addressing disparities in financial hardship. Overall, these results suggest that continuous assessment of financial hardship throughout the survivorship is essential to providing timely intervention support.

Our findings highlight the substantial impact of the psychological responses dimension on overall financial hardship over time. Noticeably, patients express financial worries 1–2 years before experiencing actual material condition hardship, which may explain the discordance between objective and subjective financial burdens found in previous studies.^36^ It is also consistent with the finding that patients are generally too overwhelmed mentally to process material condition‐related problems before they become urgent.^37^ Thus, it may be that timely psychosocial support for the most vulnerable patients is a particularly important financial hardship mitigation strategy.^38^ Our findings suggest providing targeted psychosocial support at or before formal diagnosis and 2–6 years after diagnosis when psychological distress is high compared to actual material condition hardship.

Our findings on financial hardship dynamics endorse the use of well‐timed financial navigation interventions to help patients with material condition hardship, particularly around 1 year after diagnosis, when the psychological distress level is at its lowest and patients may be more capable of addressing their financial challenges.

Dynamic patterns of financial hardship vary by cancer type, with lung cancer patients facing rapidly increasing financial pressure after diagnosis, similar to that observed elsewhere,^39^ while breast cancer patients appear to suffer less from material deprivation, especially those surviving for more than 2 years. This may be driven by different costs of cancer treatment, as a recent finding shows lung cancer patients have higher OOP spending than breast cancer patients.^4^

The subgroup analysis indicates younger patients (18–64 years old) are more prone to material condition hardship than older adults (65+ years old), who generally have more material wealth. Job loss, reduced work productivity, and reliance on employer‐provided health insurance contribute to financial struggles in young patients. Previous investigations found health insurance coverage is much higher among those 65+ years old than those 18–64 years old, about a quarter of whom are underinsured compared to those on Medicare.^40^ Although cancer registry data may underestimate non‐compliance with cancer treatments due to a lack of information about economically motivated medication nonadherence,^7^, ^11^ our analysis identified a small percentage (4.3%) of cases with missing treatments, similar to the percentage reported for patients refusing treatments to save money (5.4%).^3^

The composite index identifies a previously overlooked group of at‐risk patients who are younger, more educated, have a higher income, and have lower health insurance coverage than those currently eligible for financial assistance, who have been the focus of previous studies.^1^, ^3^, ^37^ About 40% of these high‐risk patients were not eligible for any financial assistance under current screening, highlighting the need for more comprehensive financial hardship assessments.^41^, ^42^, ^43^ Without proper identification and timely intervention, health disparities may widen due to the rising costs of cancer treatment.^44^, ^45^

This study is, to the best of our knowledge, the first attempt to construct a novel multi‐dimensional measure using existing health system data. Based on the use of available data and the potential generalizability, this composite index may have practical implications in clinical settings. One potential implication is that a financial hardship screening tool, once integrated into a health system's existing data platform, could inform financial assistance staff to reach out to patients in need in a timely manner. This information could also be shared with healthcare teams to facilitate communications on costs.^3^, ^46^ It could also allow health systems to assess financial hardship throughout a patient's disease progression and survivorship trajectory,^47^, ^48^, ^49^ potentially improving survivorship rates, patient's quality of life, and reducing treatment disparities.^15^, ^16^

Despite the strengths, our study is not without limitations. Some health systems may lack psychological distress data, hindering the use of a multi‐dimensional composite index to screen patients. However, these psychological distress data are collected by all accredited U.S. cancer centers, which provide significant portion of cancer care. Additionally, some health systems' existing electronic health records may not currently contain all relevant financial information (e.g., employment status, household savings) that directly impact patients' financial well‐being. Further, our measure of coping behaviors is limited to cancer treatment adherence and does not include other potentially important factors that will be important to assess in future research, such as medication adherence and lifestyle coping behaviors. Our low rates of treatment nonadherence are consistent with the literature finding in that the prevalence of treatment nonadherence (other than prescription medicine) is the lowest among all other financial hardship behavioral responses (Appendix 4; Table D1).^41^, ^50^ Finally, the patients excluded from the analyses due to missing data on the relevant variables were more likely to have lower incomes; more likely to identify as Hispanic, female, and African American; and less likely to have higher education levels, compared to those included in our analyses (see Appendix 3 for details). While these sample differences limit the generalizability of our results and the scalability of the algorithm, it is important to note that these same biases would likely impact current financial toxicity screening protocols as well, demonstrating the need for more comprehensive and complete assessment of relevant financial toxicity parameters. Future studies should include more diverse cancer patient cohorts for greater generalizability and to further validate our method of assessing composite financial hardship. Despite these limitations, these between‐group and between‐dimension comparisons may still inform targeted financial hardship mitigation strategies.

In summary, this study addresses the recent call for research on utilizing administrative data to better screen patients at the risk of financial hardship.^17^ This study contributes to the growing literature on cancer financial hardship by demonstrating the potential to systematically measure and track patients' financial hardship levels using existing health system data.

## AUTHOR CONTRIBUTIONS

**Wen You:** Conceptualization (lead); data curation (supporting); formal analysis (supporting); funding acquisition (lead); investigation (lead); methodology (lead); project administration (supporting); supervision (lead); validation (equal); writing – original draft (lead); writing – review and editing (equal). **Asal Pilehvari:** Formal analysis (co‐lead); investigation (supporting); project administration (co‐lead); software (equal); validation (equal); writing – original draft (supporting); writing – review and editing (equal). **Ruoding Shi:** Formal analysis (co‐lead); methodology (supporting); project administration ( co‐lead); software (equal); visualization (equal); writing – original draft (supporting). **Wendy Cohn:** Conceptualization (supporting); funding acquisition (supporting); validation (supporting); writing – review and editing (equal). **Christina Sheffield:** Conceptualization (supporting); funding acquisition (supporting); validation (supporting); writing – review and editing (equal). **Philip I‐Fon Chow:** Writing – review and editing (equal). **Becca Anne Krukowski:** Writing – review and editing (equal). **Roger Anderson:** Conceptualization (supporting); funding acquisition (supporting); writing – review and editing (equal).

## FUNDING INFORMATION

This study was supported by National Cancer Institute of the National Institutes of Health under award number: 3P30CA044579‐29S4. The content is solely the responsibility of the authors and does not necessarily represent the official views of the sponsoring agencies.

## CONFLICT OF INTEREST STATEMENT

The authors declare no conflicts of interest.

## ETHICS STATEMENT

This study was approved by the Institutional Review Board of University of Virginia (IRB‐HSR # 22686). A waiver/exempt of informed consent was granted by the IRB/Ethics Committee for this study purpose.

## CLINICAL TRIAL REGISTRATION

This study was not a clinical trial and was therefore not registered.

## PATIENT CONSENT STATEMENT

This is an observational study that uses existing data and informed consent was not needed from participants included in the study.

## PERMISSION TO REPRODUCE MATERIAL FROM OTHER SOURCES

All the analysis in this study is original and done by the Authors.

## Data Availability

The data that supports the findings of this study is not publicly available due to privacy or ethical restrictions.

## Footnotes

* We found no statistically significant differences between those 1,731 patients and the rest of the sample in terms of demographics and socio‐economic factors. The only difference we found is that those 1,731 excluded patients, on average, have more advanced stages of cancer (mean cancer stage is 3.25) as compared to those included based on this criterion (mean cancer stage is 2.9, *p*‐value is <0.000). In addition, fewer of those exclude were still alive during our data pull (*p* < 0.000).

† FA levels are determined by the following rules: 100% FA if adjusted income<100% FPL; 95% FA if adjusted income is between 100.1%–110% FPL; 80% FA if adjusted income is between 110.1%–133% FPL; 55% FA if adjusted income is between 133.1%–167% FPL; 30% FA if adjusted income is between 167.1%–200% FPL; 60% charity if adjusted income is between 201%–300% FPL; 50% charity if adjusted income is between 301%–400% FPL.

‡ The Rapid Quality Report System was launched in 2011 and was replaced by Rapid Cancer Reporting System (RCRS) in 2020 as the new data submission system to the Commission on Cancer's National Cancer Database. See details in: https://www.facs.org/quality‐programs/cancer/ncdb/rcrs.

§ Research shows that 10,000 simulations are sufficient to approximate the impact of different weight choices on the composite index.^24^ This feature of SMAA makes it well‐suited in health outcome evaluations such as ours, because individual heterogeneity is an important issue in the health context. ^25^

¶ Note: We follow the current literature in defining the financial hardship, Which includes not just objective financial distress but also subjective distress caused by cancer diagnosis and treatment (i.e., distress caused by direct financial worries and treatment‐related worries).^29^

## APPENDIX 1 TOTALLY FUZZY AND RELATIVE APPROACH

### 1.1

Deutsch and Silber^51^ provide a detailed description of the fuzzy set approach. The objective of this method is to transfer indicators on different scales to a score between 0 and 1 depending on variable types as follows:

(1) If the indicator was dichotomous (e.g., if have insurance), its fuzzy membership score was either 0 or 1 depending on which state contributes to a higher financial hardship (e.g., 1 = uninsured and 0 = insured).

(2) Ordinal variables were rearranged and linearly scored within the interval of [0,1] to reflect the rank of individuals in terms of the level of each indicator. ** Take education as an example for ordinal variables, 0 = completed college and above, 0.25 = some college, 0.5 = completed high school, 0.75 = some high school, 1 = less than high school. One example is provided in Table 1 for transforming FA eligibility status.

(3) For continuous variables, Cheli, and Lemmi^20^ proposed a totally fuzzy and relative approach (TFR). Take household income as an example, the basic idea of TFR is to assign the membership based on the ranking of the individual's income in the sample. However, it is not reasonable to rank the whole sample because once the household income exceeds a threshold (i.e., 400% FPL), the household should not be considered as income deprived. That is.

1. If patient i’s household income is above or equal to 400% FPL, the degree of membership is $u_{m}\left(i\right)=0.$
2. If the person’s household income is below 400% F.P., the degree of membership is $u_{m}\left(i\right)=u_{m-1}\left(j\right)+\left(F_{m}\left(u\right)-F_{m-1}\left(u\right)\right)/\left(1-F\left(u=0\right)\right)$.

Where $F_{m}\left(.\right)$ is the sample cumulative distribution function of household income, and m is the rank for a patient i below the threshold of 400% FPL. $F\left(u=0\right)$ is the cumulative probability for $u_{m}\left(i\right)=0$. To calculate the degree of membership, we ranked all patients below the threshold in an increasing order with respect to risk of financial hardship (e.g., in a decreasing order of income). Starting from the first patient below the threshold who is least poor, the membership score of patient i in rank m was calculated as a sum of the membership score of patient j in rank m−1 plus the normalized inclement of cumulative sample distribution from rank *m* to rank m−1.^20^, ^50^

Aggregating indicators within a dimension within each dimension, the K indicators need to be aggregated into one deprivation score for patient i. Widely used methods in fuzzy set approach literature are intersection and union:

(Intersection)
$$u\left(i\right)=\min\left(u\left(x_{1i}\right),u\left(x_{2i}\right),u\left(x_{2i}\right),\ldots ,u\left(x_{\mathrm{Ki}}\right)\right))$$

(Union)
$$u\left(i\right)=\max\left(u\left(x_{1i}\right),u\left(x_{2i}\right),u\left(x_{2i}\right),\ldots ,u\left(x_{\mathrm{Ki}}\right)\right))$$

The third option is to calculate the score as a weighted average of values of indicators within a domain, and previous studies have suggested different weighting methods, such as equal weights (average), from expert opinions or nominalized by relatively deprivation (deprivation‐weighted average).

In this study, we used four fuzzy set aggregators: intersection, union, equal weights and the weight as an inverse function of the average degree of deprivation in the population. The last weighting method assumes that the lower the frequency of poverty according to a given indicator, the greater the weight this indicator will receive. The weight of indicator k can be specified as follows:

$$w_{k}=\ln\left(1/\bar{u}\left(x_{k}\right)\right)$$

where $\bar{u}\left(x_{k}\right)=\frac{1}{n}\sum_{i=1}^{n}u\left(x_{\mathrm{ki}}\right)$ reflects sample mean deprivation score with respect to indicator k. However, since the calculated weights are unlikely to add up to 1, we need to adjust the denominator to get the weighted average score as the deprivation score in one dimension.

$$u\left(i\right)=\frac{\sum_{k=1}^{K}u\left(x_{\mathrm{ki}}\right)\cdot w_{k}}{\sum_{k=1}^{K}w_{k}}$$

This equation ensures that the lower the sample mean score of a given deprivation indicator, the greater the weight this indicator will receive for a person who is indicated poor by this indicator. The intuition behind this weighting method is that if most patients are not deprived in this indicator, a particular patient who is poor in terms of this indicator will receive a higher weight reflecting the concept of relative poverty.

## APPENDIX 2 PRINCIPAL COMPONENT ANALYSIS ON PSYCHOLOGICAL RESPONSES DIMENSION OF THE FINANCIAL TOXICITY

**TABLE B1:** Correlation matrix between the original indicators in support screen and the principal component.

Survey items	Extracted principal components (P.C.)			
	First	Second	Third	Fourth
Highly correlated with emotional distress				
Feeling anxious or afraid	0.76	−0.15	−0.23	−0.28
Worry about physical appearance	0.55	0.02	0.06	0.07
Feeling sad or depressed	0.83	−0.18	−0.12	−0.18
Feeling my life has lost meaning/purpose	0.65	−0.17	−0.11	−0.05
Worry about the future	0.79	−0.1	−0.25	−0.06
Managing your emotions (feeling hopeless, angry, etc.)	0.78	−0.17	−0.19	−0.14
Fear of treatment	0.58	−0.04	−0.28	0.42
Highly correlated with financial‐related distress				
Finances or health insurance	0.51	0.85	0.06	−0.08
Highly correlated with energy deprivation				
Fatigue/Feeling Tired	0.65	−0.16	0.73	0.01
Highly correlated with treatment‐related worry				
Understanding my illness/treatment options	0.53	−0.05	−0.07	0.64
Rest				
Transportation or Lodging	0.39	0.23	−0.13	0.34
Communicating healthcare wishes to the healthcare team	0.39	−0.01	−0.05	0.4
Alcohol, street drugs, and prescription drug use	0.18	0	−0.01	0.19
Talking with my family about my illness	0.46	−0.04	−0.09	0.15
Making appointments/keeping appointments straight	0.43	0.02	−0.05	0.28

*Note*: This table reports correlation coefficients between original survey responses with four extracted principal components. Shading indicates the highest correlations (and p>0.5) among extracted components for each survey question.

## APPENDIX 3 WHO ARE THOSE WITH MISSING DATA

**TABLE C1:** Summary statistics comparing excluded sample due to missingness and the analytical sample.

	Sample with missing data	Analytical sample	Test
*N*	741	303	
Income (SD)	60,381.4	69,513.3	0.04
	(50,782.5)	(54,168.2)	
Age categories			
18–44	29 (4.1%)	20 (6.6%)	0.277
45–64	282 (39.5%)	125 (41.3%)	
65+	403 (56.4%)	158 (52.1%)	
Marital status			
Married	399 (55.9%)	208 (68.6%)	<0.001
Not married	315 (44.1%)	95 (31.4%)	
Ethnicity			
Hispanic	22 (3.1%)	1 (0.3%)	0.019
Non‐Hispanic	687 (96.9%)	302 (99.7%)	
Gender			
Female	596 (83.4%)	224 (73.9%)	<0.001
Male	119 (16.6%)	79 (26.1%)	
Race			
African American	56 (7.9%)	10 (3.3%)	0.021
Other	25 (3.5%)	7 (2.3%)	
White	628 (88.6%)	285 (94.4%)	
Living status			
Deceased	64 (9.0%)	68 (22.7%)	<0.001
Alive	649 (91.0%)	231 (77.3%)	
Education			
High school degree	63 (42.9%)	24 (8.3%)	0.002
Less than high school	11 (7.5%)	69 (23.8%)	
Some college or higher	73 (49.7%)	197 (67.9%)	
Cancer Stage			
Early to mid‐stage (stage 0–2)	481 (84.1%)	158 (54.5%)	<0.001
Late stage (stage 3 and 4)	91 (15.9%)	132 (45.5%)	

The Table C1. presents the comparison between those 741 patients excluded from the analysis and those 303 patients included in our analytical sample. As shown in Figure 1 of the paper, among those 741 patients, 354 of them have missing relevant medical record data, 385 of them do not have complete information in support screen psychosocial response data, and the remaining 2 patients are not in the cancer registry data. Therefore, this appendix section aims at comparing those two samples and provides evidence of who are those with missing data and how does it mean for our current study result interpretation.

As expected, those with missing data in the existing health system databases have relatively lower income, are less likely to be married, and are less likely to have higher education levels. They also are more likely to be Hispanic, female, or African American and be diagnosed with early‐stage cancers.

However, we do want to highlight the fact that existing financial toxicity screening not only uses the same existing data with similar types of missing patterns, which is another important concern about the current financial toxicity screening protocols. Our study is hopefully a first step in understanding the limitations of single‐dimension, single‐variable, arbitrarily‐set frequency of existing screening given the limited existing health system data. Another important and relevant point is that recent literature has placed increasing attentions on those ‘near‐poor’ cancer patients whose income is between 100% and 200% of the Federal Poverty Line. Those individuals are not eligible for Medicaid or other low‐income subsidies and are the most vulnerable subgroups for financial toxicity. Our sample covers those ‘near‐poor’ populations who can benefit most from the comprehensive screening.

## APPENDIX 4 TREATMENT NONADHERENCE BREAKDOWNS BY CANCER TYPES

**TABLE D1:** Treatment nonadherence by cancer types.

Treatment nonadherence	Breast	Colon	Lung	Other	Total
Immunotherapy	1	0	0	0	1
Radiation	3	0	1	0	4
Chemotherapy	3	4	3	0	10
Hormonotherapy	3	0	0	1	4
